# Etymologia: Plague

**DOI:** 10.3201/eid2401.ET2401

**Published:** 2018-01

**Authors:** Ronnie Henry

**Keywords:** etymologia, plague, Yersinia pestis, bacteria

## Plague [plāg]

**Figure 1:**
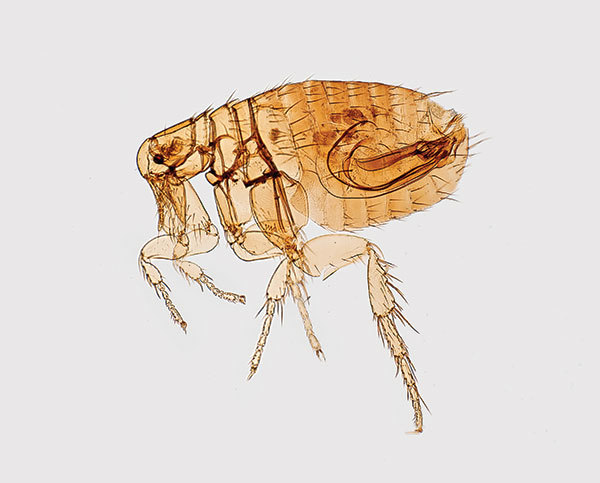
Digitally colorized scanning electron microscopic image of a flea. Fleas are known to carry a number of diseases that are transferable to humans through their bites, including plague, caused by the bacterium *Yersinia pestis*. Photo: Centers for Disease Control and Prevention (CDC), Janice Haney Carr.

**Figure 2:**
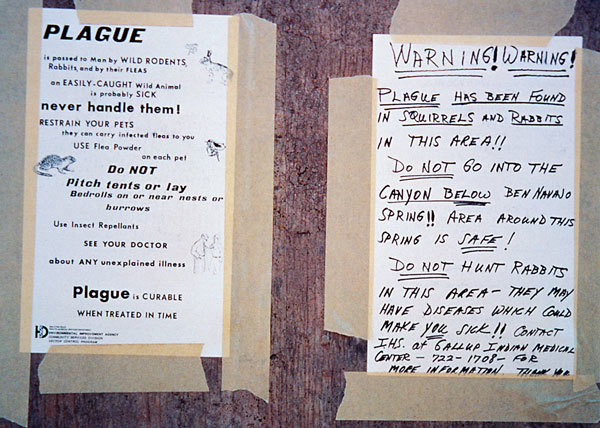
Plague warning signs posted in regions where plague has been discovered. In remote areas with little human habitation, the most appropriate action may be to post signs on the roads entering the epizootic area to warn people, and provide information on personal protection and plague prevention. Photo, CDC, 1993.

Plague (from the Latin *plaga*, “stroke” or “wound”) infections are believed to have been common since at least 3000 bce. Plague is caused by the ancestor of current *Yersinia* (named for Swiss bacteriologist Alexandre Yersin, who first isolated the bacterium) *pestis* strains (Figure 1). However, this ancestral *Y. pestis* lacked the critical *Yersinia* murine toxin (*ymt*) gene that enables vectorborne transmission. After acquiring this gene (sometime during 1600–950 bce), which encodes a phospholipase D that protects the bacterium inside the flea gut, *Y. pestis* evolved the ability to cause pandemics of bubonic plague. The first recoded of these, the Justinian Plague, began in 541 ace and eventually killed more than 25 million persons (Figure 2).
